# Overweight at four years of age in a Swedish birth cohort: influence of neighbourhood-level purchasing power

**DOI:** 10.1186/s12889-016-3252-1

**Published:** 2016-07-11

**Authors:** Josefine Roswall, Gerd Almqvist-Tangen, Anders Holmén, Bernt Alm, Stefan Bergman, Jovanna Dahlgren, Ulf Strömberg

**Affiliations:** Department of Paediatrics, Halland Hospital, Halmstad, Sweden; Department of Paediatrics, Institute of Clinical Sciences at the Sahlgrenska Academy, University of Gothenburg, Gothenburg, Sweden; Department of Research & Development, Region Halland, Halmstad, Sweden; Child Health Care Unit, Region Halland, Kungsbacka, Sweden; Primary Health Care Unit, Department of Public Health and Community Medicine, Institute of Medicine, The Sahlgrenska Academy, University of Gothenburg, Gothenburg, Sweden; Research and Development Centre Spenshult, Halmstad, Sweden; Health Metrics Unit, Department of Public Health and Community Medicine, Institute of Medicine, The Sahlgrenska Academy, University of Gothenburg, Gothenburg, Sweden

**Keywords:** Childhood overweight, Prevention strategy, Economic background

## Abstract

**Background:**

A number of child/parental factors have been shown to be significant predictors of childhood overweight, although a better understanding of possible contextual influences of neighbourhood-level characteristics might provide new insights leading to tailored, targeted interventions. The aim of this study was to explore the impact of neighbourhood purchasing power and its relationship with other known risk factors related to childhood overweight in a prospective birth cohort.

**Methods:**

A prospective, population-based, birth-cohort study was conducted in south-western Sweden, comprising 2,666 infants born in 2007–2008. Childhood overweight was assessed by body mass index (BMI) data from follow-up examinations at four years of age (*n* = 2,026) and overweight defined according to the International Obesity Task Force. Using logistic regression analysis, the influential child/parental predictors were identified from the candidate predictors, *viz*. child’s gender, as well as birth weight adjusted for gestational age and parental factors at recruitment, including maternal smoking status, maternal BMI (before pregnancy), paternal BMI and parental educational level. The children’s residential parishes at follow-up were stratified by parish-level household purchasing power (<10 %, 10–19.9 %, 20–29.9 % and ≥30 % of *all* resident families with low purchasing power) and the “contextual” influence was analysed. In each such neighbourhood stratum, the adjusted overweight ratio (AOR), i.e. the ratio between the observed number of overweight children and the expected number, taking account of the influential child/parental predictors, was estimated.

**Results:**

The prevalence of overweight at four years of age was 11.9 %. In the economically strongest neighbourhoods (i.e. <10 % of resident families with low purchasing power), the AOR was 0.60 (95 % confidence interval (CI): 0.34–0.98). The corresponding empirically Bayes-adjusted AOR was 0.73 (95 % CI: 0.46–1.02; 97 % posterior probability of AOR <1). In the other neighbourhood strata, the statistical evidence of a deviant AOR was weaker.

**Conclusion:**

The economically strongest neighbourhoods had a lower prevalence than expected of overweight at four years of age. This finding should prompt studies to acquire more knowledge of potentially modifiable factors that differ between neighbourhoods and are related to childhood overweight, providing a basis for tailored, targeted interventions.

## Background

Childhood overweight and obesity have been increasing during the last 30 years. Among preschool children in Europe, overweight rates vary from 11 % in Romania to 30 % in Spain [1, 2]. In Sweden, 15.6 % of seven to nine year olds have been found to be overweight [3] Childhood obesity shows a strong tracking into adulthood, and many children already become obese in early childhood [4, 5]. Life-time health risks, such as hyperlipidaemia, type 2 diabetes and heart disease, are related to childhood overweight [6]. Understanding factors influencing the risk of developing childhood overweight and obesity is important in order to tailor early intervention strategies.

Increasing evidence suggests that intrauterine and early life factors influence the risk of developing obesity. Both over- and under nutrition during uterine life is known to lead to an increased risk of obesity and subsequent metabolic health risks [7–10]. Smoking during pregnancy and parental obesity and educational level has also been related to increased risk of overweight and obesity [11–13].

Associations between low socio-economic status (SES) and childhood obesity risk have been observed in several studies [11, 14]. During the beginning of the obesity epidemic, the increase in childhood obesity rates was reported to affect all SES groups [15]. Recent reports from some high-income countries now indicate a promising levelling-off in childhood overweight and obesity rates, but unfortunately only in some countries and not in others [16, 17]. Increasing disparity has been described between urban and rural areas, showing decreasing obesity rates in urban but not rural areas [3]. On the other hand, obesity rates are still increasing in many developing countries presenting with a differential influence of geographical and socio-economic factors, with the greatest increase in obesity rates in urban areas in developing countries [18].

Previous research has found that child overweight and obesity are independently and negatively influenced by neighbourhood SES [19]. Health-related problems have been reported to be strongly associated with the social characteristics of communities and neighbourhoods [19, 20]. One influential contextual factor that has been identified relates to food and grocery stores; whether they are located nearby and whether they offer a variety of healthy food choices at a reasonable cost [21]. Observations based on aggregated data on food purchases and household SES indicate that poorer quality diets are more frequently consumed in households with limited economic means [22]. Studies have shown considerable ethnic, socio-economic and behavioural disparities in childhood obesity and some studies have attempted to examine the impact of different neighbourhood conditions, such as safety, built-up environment or access to healthy food choices, on childhood obesity [23].

A better understanding of possible contextual influences of neighbourhood-level characteristics might provide new insights leading to tailored, targeted interventions. The aim of this study was to explore the impact of neighbourhood purchasing power and its relationship with other known risk factors in relation to childhood overweight in a prospective birth cohort.

## Methods

The present study is part of a larger Swedish project called the “Halland Health and Growth Study” (H^2^GS). The main goal of the H^2^GS is to increase our understanding of the concept of child health and growth from a parental perspective, focusing on parental needs, and a medical/social perspective, elucidating risk factors for growth disturbances. The project was approved by the Research Ethics Committee at Lund University. Written consent was obtained from the parents of the infants involved.

### Study design

The H^2^GS was designed as a prospective, population-based, birth-cohort study that recruited newborns and their families in the region of Halland, south-western Sweden, between 1 October 2007 and 31 December 2008, with 2,666 of a total of 3,860 newborns included. Parents and infants were recruited at first regular visit to child health care clinic at around 1 week of age. Written informed consent was collected from the infants parents. Parents filled in questionnaires regarding background data, food and lifestyle at newborn, 4, 12, 18, 24, 36 and 48 M of age. Height, weight, head and waist circumference was measured by trained child health care nurses at each of these time points. The study protocol, recruitment strategy and the representativeness of the sample have been reported in detail elsewhere [24].

### Outcome: overweight at four years of age

Follow-up data for the cohort children have been registered at one, four, six, 12 and 18 months, as well as at two, three and four years of age. We focus here on outcome data on body mass index (BMI) at four years. In all, 2,026 children (1,013 boys and 1,013 girls) with outcome data were included. Their median age at examination was 4.0 years (range: 3.6–5.0 years; 11 children were older than 4.5 years). The examinations were performed during the calendar period 18 August 2011 to 30 October 2013.

The reported BMIs were transformed to gender- and age-specific standard deviation scores (SDS), using the estimated mean and SD functions based on Swedish reference data [25]. Hence, SDS equals the difference between the observed and estimated mean BMI divided by the SD, with regard to gender and age. Taking into account the internationally established reference BMI values presented by Cole [26], overweight at 4.0 years of age was defined as having an SDS of >1.19 for boys or an SDS of >1.11 for girls. These SDS cut-offs also classified overweight in accordance with Cole’s reference BMI values for the children with ages at examination deviating from 4.0 years, within the age range 3.6–5.0 years (due to the adjustment for age incorporated in the SDS calculation).

### Candidate child/parental predictors of overweight

Child gender was a candidate predictor of overweight, indicated by the fact that differential gender-specific cut-off points for SDS were derived from Cole’s international reference values (SDS >1.19 for boys and SDS >1.11 for girls correspond to *expected* proportions of 11.7 % and 13.3 % respectively in the Swedish reference population) [25]. Another predictor was birth weight adjusted for gestational age, with data obtained from medical records (not available for six children delivered outside the region of Halland). More precisely, the children were classified as either born large for gestational age (LGA) if >2 SDS (*n* = 66) or normal birth weight (*n* = 1937) respectively, according to Swedish reference standards [27]. Candidate parental predictors included well known risk factors such as mother’s smoking status (no, yes), mother’s BMI before pregnancy (≤25, >25-30, >30), father’s BMI (≤25, >25-30, >30) and parental educational level (none post-secondary, one post-secondary, both post-secondary). [11, 28, 29]. Data on the candidate parental predictors were self-reported at the first visit at the child health care center (usually one week after childbirth), i.e. when the first questionnaire was filled out. These data are not complete for the families of the 2,026 study children (numbers of missing values: mother smoking, *n* = 91; mother’s BMI, *n* = 137; father’s BMI, *n* = 329; and parental educational level, *n* = 207).

The frequency distributions of the candidate predictors are given in Table 1. The prevalence of smoking mothers was 5.1 % (disregarding mothers with missing values). The prevalence of obese mothers (BMI >30) before pregnancy was 7.8 %. The prevalence of obese fathers was 7.7 %. Thirty-five per cent of the families had a “low” educational level, i.e. none of the parents had attained a post-secondary education.

**Table 1:** Associations between overweight at four years of age and the candidate predictors

		Univariate models		Multivariate model^e^	
*Candidate predictors*	n (% overweight)^a^	OR (95 % CI)^d^	*P*	OR (95 % CI)^d^	*P*
Child variables					
*Gender*			*0.07*		*0.083*
Female	1013 (13.2)	1.28 (0.98–1.67)		1.32 (0.97–1.83)	
Male	1013 (10.7)	1.00 (reference)		1.00 (reference)	
*Large for gestational age (LGA)*			<*0.001*		*0.003*
No^b^	1954 (11.5)	1.00 (reference)		1.00 (reference)	
Yes	66 (27.3)	2.90 (1.66–5.07)		2.74 (1.42–5.28)	
Parental variables^c^					
*Mother smoking*			*0.05*		Not included
No	1836 (11.6)	1.00 (reference)			
Yes	99 (18.2)	1.69 (1.00–2.88)			
*Mother’s BMI before pregnancy*			*<0.001*		*0.01*
≤25	1407 (10.0)	1.00 (reference)		1.00 (reference)	
25–30	334 (16.8)	1.81 (1.29–2.53)		1.56 (1.06–2.29)	
30+	148 (19.6)	2.19 (1.41–3.40)		1.87 (1.14–3.09)	
*Father’s BMI*			*<0.001*		*<0.001*
≤25	866 (7.4)	1.00 (reference)		1.00 (reference)	
25–30	701 (13.0)	1.87 (1.34–2.62)		1.84 (1.30–2.60)	
30+	130 (25.4)	4.26 (2.67–6.82)		3.49 (2.11–5.75)	
*Parental educational level*			*0.001*		*0.03*
None post-secondary	641 (15.0)	2.03 (1.41–2.92)		1.69 (1.13–2.53)	
One post-secondary	578 (11.4)	1.48 (1.00–2.19)		1.29 (0.84–1.98)	
Both post-secondary	600 (8.0)	1.00 (reference)		1.00 (reference)	

^a^Study children categorised according to each candidate predictor. The total number of children in the birth cohort with data on overweight at four years of follow-up = 2,026. The number of missing values for candidate predictors: LGA, *n* = 6; mother smoking, *n* = 91; mother’s BMI, *n* = 137; father’s BMI, *n* = 329; parental educational level, *n* = 207

^b^This category includes 23 SGA (small for gestational age) children (none of these children was overweight at four years)

^c^Questionnaire data obtained at recruitment

^d^Estimated odds ratio with 95 % confidence interval

^e^Backward selection of the candidate predictors (*P* for exclusion >0.10). No significant interaction effects between the selected variables. Number of children with complete data for the selected variables = 1,643 [818 boys (82 with overweight, 10.0 %) and 825 girls (101 with overweight, 12.2 %)]

### Neighbourhood-level characteristics

**Fig. 1:**
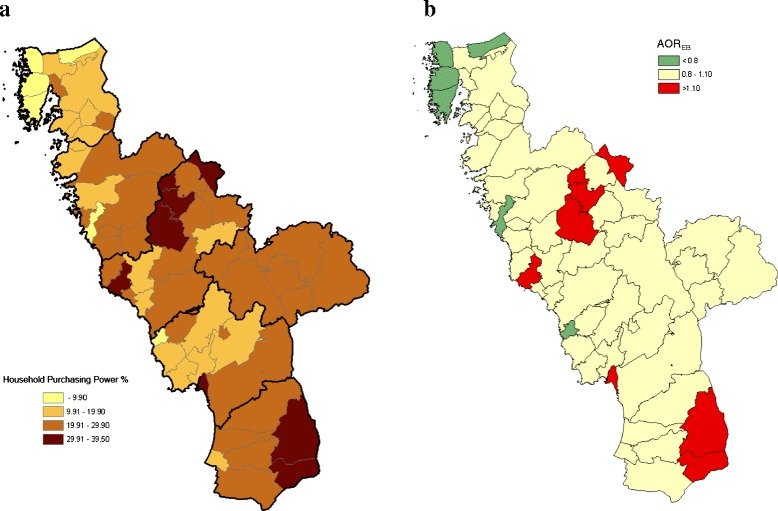
Geo-map of neigbourhood purchasing power. **a** Geo-map of household purchasing power for the 58 parishes in the County of Halland. The residential areas (parishes) were classified into <10 %, 10–19 %, 20–29 % and ≥ 30 % based on this indicator [according to the Swedish standard, corresponding to ≤ USD 19, 500 annual household purchasing power among all resident families with at least one child (≤19 years old: family with the same residential address)]. Household purchasing power was defined as total family disposable income adjusted for the composition of the family (number of adults and children). **b** The corresponding geo-map, based on the grouping of the parishes according to neighbourhood-level purchasing power (a), for relative risks of overweight at four years of age, denoted AOR_EB_, by stratification of child’s gender, LGA, mother’s BMI before pregnancy (≤25, 25–30, 30+), father’s BMI (≤25, 25–30, 30+) and parental educational level (none post-secondary, one post-secondary, both post-secondary)

Statistics Sweden provided parish-level data from 2010, relating to the indicator we were considering, *viz*. the proportion of families with low household purchasing power among *all* resident families with at least one child ≤19 years old; family with the same residential address. Household purchasing power was calculated as total disposable family income adjusted for the composition of the family (number of adults and children), while low household purchasing power was defined according to the Swedish standard, corresponding to ≤19,500 USD annually. The parishes were classified into <10 %, 10–19.9 %, 20–29.9 % and ≥30 %, based on the indicator reflecting neighbourhood purchasing power (Fig. 1a). The same indicator and classification have been used in a previous report on breastfeeding based on this birth cohort [30].

We aimed to geo-code the study children according to their residential parishes at the follow-up examination and we were able to match the study children to the national population registry in May 2014. As a result, each child was geo-coded with respect to his/her residential parish nationally registered in May 2014 (58 parishes in the region). We were not able to geo-code six study children, as they had moved away from the region of Halland.

### Statistical methods

We analysed associations between overweight at four years of age and the candidate predictors using logistic regression. Firstly, univariate analyses for each candidate predictor were carried out. A multiple logistic regression model was then fitted by backward selection of the candidate predictors (*P*-value for exclusion >0.10) and possible interaction effects were evaluated. Only significant interactions (*P* <0.05) were included [31].

The possible contextual influence of neighbourhood purchasing power was addressed tentatively by descriptive statistics in each neighbourhood-level stratum. We performed a multi-level analysis in order to estimate the influence of neighbourhood purchasing power. Multi-level modelling distinguishes individual and neighbourhood levels of information in a model [32]. The multi-level analysis estimated, for each group of residential areas, the *adjusted overweight ratio* (AOR), i.e. the ratio between the observed number of overweight children in a given neighbourhood stratum and the expected number for the total cohort with stratification for the influential child/parental predictors. The empirical Bayes-adjusted estimate of AOR (denoted AOR_EB_) was calculated by employing a hierarchical Bayesian model, using a prior Gamma model for the neighbourhood-level AORs (AOR_*i*_ ~ Gamma(α, β), *i* =1, 2, 3 and 4; corresponding to the four neighbourhood strata defined above) [33]. Bayesian smoothing of this kind generally yields “shrinkage” of the conventional AORs towards the expected average (i.e. AOR = 1), which can be justified statistically [32].

For comparisons, the *crude overweight ratio* (COR) in each neighbourhood stratum was estimated analogously, but without stratification for the influential child/parental predictors. The empirical Bayes-adjusted COR is denoted COR_EB_. Moreover, we checked how sensitive the results were due to missing values for the influential child/parental predictors by 1) imputing the missing values, using the iterative Markov Chain Monte Carlo method with fully conditional specification, and 2) computing AORs as well as AOR_EB_:s with complete data.

The statistical computations were performed using IBM SPSS 20.0.2 and, for the multi-level analyses, the Rapid Inquiry Facility free software [34].

## Results

108 boys (10.7 %) and 134 girls (13.2 %) were overweight at four years. Each candidate predictor showed a marked effect on overweight at four years (Table 1). The final multiple logistic regression model excluded the candidate predictor of *mother smoking* (Table 1), due to confounding by other influential predictors. There were no significant interaction effects.

The prevalence of overweight among children in the study living in the economically strongest neighbourhoods (i.e. less than 10 % of all resident families with low purchasing power) was 6.8 %, as compared to 12.8–13.5 % in the other neighbourhood purchasing power strata (Table 2). The fact that the distributions of the influential parental predictors also varied across these neighbourhood strata should be taken into account. Furthermore, descriptive statistics indicated varying associations between child/parental predictors and overweight across the neighbourhood purchasing power strata (Table 2). For example, indications of clear associations between father’s BMI and overweight at four years were seen in all neighbourhood strata except the economically strongest neighbourhoods. The number of observations in the poorest neighbourhoods was relatively small (*n* = 104).

**Table 2:** Number of study children (n) and percentage with overweight (OW) at four years of age in four groups of residential areas categorised by the contextual variable of neighbourhood purchasing power, totally and stratified for each selected predictor

	*Neighbourhood purchasing power* ^a^							
	<10		10–19.9		20–29.9		30+	
	*n*	OW (%)	*n*	OW (%)	*n*	OW (%)	*n*	OW (%)
Total^b^	310	6.8	746	13.4	860	12.4	104	13.5
*Child’s gender*								
Male	159	7.5	359	10.3	446	12.1	45	11.1
Female	151	6.0	387	16.3	414	12.8	59	15.3
*LGA* ^c^								
No	299	6.4	721	12.8	828	12.0	101	13.9
Yes	11	18.2	24	33.3	28	28.6	2	0.0
*Mother’s BMI before pregnancy* ^c^								
≤25	227	3.7	535	11.8	582	10.7	67	11.9
25–30	57	12.3	120	17.5	139	17.3	18	22.2
30+	18	18.8	48	18.8	77	19.5	7	28.6
*Father’s BMI* ^c^								
≤25	147	5.4	330	8.5	349	6.9	38	10.5
25–30	110	6.4	256	14.1	298	14.4	35	14.3
30+	15	6.7	46	28.3	57	28.1	10	30.0
*Parental educational level* ^c^								
None post-secondary	59	6.8	193	14.0	348	16.7	39	17.9
One post-secondary	88	8.0	242	12.0	218	12.4	29	10.3
Both post-secondary	144	5.6	235	12.8	194	3.1	24	16.7

^a^Proportion (%) of families with low purchasing power (according to Swedish standards; <19,500 USD annual income) among all resident families with at least one child (up to 19 years old) in a neighbourhood area (parish)

^b^The total number of children in the birth cohort with data on overweight at four years of follow-up = 2,026; it was possible to geo-code 2,020 of these children

^c^Predictors with missing data, cf. Table 1

The multi-level analysis, which accounts for the influential child/parental predictors, yielded AOR_EB_ = 0.73 (95 % confidence interval: 0.46-1.02) for the economically strongest neighbourhoods (Table 3). In other words, among the children living in the economically strongest neighbourhoods, 27 % fewer children than expected were overweight and the posterior probability of AOR <1 was 0.97 [35]. In the other neighbourhood strata, the statistical evidence of deviant AORs was weaker (Table 3, Fig. 1b).

**Table 3:** Observed (Obs) and expected (Exp) numbers of children with overweight at four years of age, together with the results from the multi-level analysis. Moreover, an example of results obtained by imputing missing values is presented in light grey

	Neighbourhood level without adjustments				Neighbourhood level with adjustments^c^				Neighbourhood level with adjustments^c^			
	Obs	Exp	COR (95 % CI)^a^	COR_EB_ (95 % CI)^b^	Obs	Exp	AOR (95 % CI)^d^	AOR_EB_ (95 % CI)^e^	Obs	Exp	AOR (95 % CI)^d^	AOR_EB_ (95 % CI)^e^
*Neighbourhood purchasing power* ^f^									*Example of results with missing values imputed:*			
<10	21	37.0	0.57 (0.35–0.87)	0.67 (0.46–0.93)	16	26.6	0.60 (0.34–0.98)	0.73 (0.46–1.02)	21	33.9	0.62 (0.38–0.95)	0.74 (0.51–0.99)
10–19.9	100	89.7	1.11 (0.91–1.36)	1.09 (0.90–1.31)	74	66.6	1.11 (0.87–1.39)	1.09 (0.86–1.34)	100	87.3	1.15 (0.93–1.39)	1.11 (0.91–1.33)
20–29.9	107	102.6	1.04 (0.86–1.26)	1.03 (0.85–1.23)	81	79.8	1.01 (0.81–1.26)	1.01 (0.81–1.23)	107	107.8	0.99 (0.82–1.20)	0.99 (0.81–1.17)
30+	14	12.6	1.11 (0.61–1.86)	1.03 (0.67–1.48)	12	9.3	1.28 (0.66–2.24)	1.13 (0.69–1.62)	14	13.1	1.07 (0.59–1.80)	1.01 (0.66–1.40)

^a^Crude overweight ratio = Obs/Exp (95 % confidence interval)

^b^Empirical Bayes-adjusted COR (95 % confidence interval)

^c^Data were stratified by child’s gender, LGA, mother’s BMI before pregnancy (≤25, 25–30, 30+), father’s BMI (≤25, 25–30, 30+) and parental educational level (none post-secondary, one post-secondary, both post-secondary), in order to adjust for potential confounding across the neighbourhoods

^d^Adjusted overweight ratio = Obs/Exp (95 % confidence interval)

^e^Empirical Bayes-adjusted AOR (95 % confidence interval)

^f^Proportion (%) of families with low purchasing power (according to Swedish standards; <19,500 USD annual income) among all resident families with at least one child (up to 19 years old) in a neighbourhood area (parish)

By imputing the missing values, the estimates changed only marginally and (as could be expected) the precision improved somewhat (Table 3). The results were consistent across repeated imputations (data not shown).

## Discussion

In this study, we have shown an independent effect of neighbourhood area purchasing power on the prevalence of early childhood overweight. The economically strongest neighbourhoods presented a clearly reduced risk of overweight in early childhood and the associations between individual known influential factors differed according to neighbourhood area economic status. The economically strongest neighbourhood areas appeared to be protected against the effect of overweight in the father; the overall effect of father’s weight status was almost abolished. This was contrary to the finding that paternity weight status, , still strongly influences risks in the poorer neighbourhoods.

It is worth noting that a high educational level had an impact on childhood overweight in some but not all areas according to neighbourhood purchasing power (Table 2). Generally, our data indicated higher levels of overweight in the mean-/low-income areas than in the affluent neighbourhoods, regardless of educational level. This should be an insight of concern, emphasising the complexity of the socio-economic influence on an increased risk of obesity. Sweden is known to have a high standard of living and a highly educated population. The state offers parents a well-developed parental leave programme and free child health care. The Swedish Child Health Care/Servíces are free and voluntary and encompass almost 100 % of the families with preschool children. The basic part of health supervision comprises programmes for development assessment, immunisation and health examination (the National Board of Health, 2014). The country ranks among the top countries in the Organisation of Economic Co-operation and Development (OECD) Better Life Index [36]. This study indicates that, despite the previous facts, neighbourhood area inequalities still persist and urgently need to be addressed. In our study, neighbourhood area purchasing power was more strongly related to overweight than parental educational levels. This was also found when it came to formula feeding [37] and indicates a need for targeted, tailored intervention, addressing the direction of resources towards areas with increased needs. In this study, we include several different known risk factors for childhood overweight and obesity, as well as different markers of socio-economic status at individual and neighbourhood levels, possibly describing different frameworks of influence, where parental education has been more related to knowledge and beliefs and the neighbourhood purchasing power to access resources [38, 39]. Further studies are needed to address area-specific societal differences affecting population health at neighbourhood level.

Lifestyle factors and behaviours that are adopted very early in life tend to persist throughout life [24]. Studies show that investing in quality programmes and services that support the family’s earliest development produces a higher rate of return than investments made later in life [27].

Neighbourhood data, reflecting contextual SES, were available at parish level. It could not be assumed that each parish was a homogeneous spatial area in terms of SES. Nevertheless, it emerged that the method, when applied to categorising neighbourhood purchasing power, based on parish-level data, revealed a contextual effect. It is possible that other spatial areas might have revealed a more pronounced contextual effect. We considered neighbourhood purchasing power as the primary indicator of neighbourhood socio-economy. The indicator takes only resident families with at least one child (≤19 years of age) into consideration and the elderly population is ignored.

The socio-economic statistics applied here were from May 2014 (when the children were 5.3- 6.5 years of age instead of four years), which could be seen as a weakness, but this was only a minor concern, as the neighbourhood characteristics appeared to be stable over the years [14]. Approximately 12 % of four-year-old children in our study presented with overweight according to the IOTF, but the rates ranged from 6.8 to 13.5 %, depending on neighbourhood area purchasing power.

## Conclusion

A lower than expected prevalence of overweight at four years of age in the economically strongest neighbourhoods suggests a contextual influence, even though residual confounding of uncontrolled parental/child factors cannot be ruled out. This finding should prompt studies to acquire more knowledge about potentially modifiable factors that differ between neighbourhoods and are related to childhood overweight, providing a basis for tailored, targeted interventions.

## Abbreviations

AOR, adjusted overweight ratio; AOR,_EB_ The empirical Bayes-adjusted estimate of adjusted overweight ratio; BMI, body mass index; CI, confidence interval ; COR, crude overweight ratio; H^2^GS, Halland Health and Growth Study; LGA, large for gestational age; OECD, Organization of Economic Co-operation and Development; SDS, standard deviation scores; SES, socio-economic status.
